# The Effect of Calcium With or Without Calcitriol Supplementation on Renal Function in Patients With Hypovitaminosis D and Chronic Kidney Disease

**DOI:** 10.5812/numonthly.13381

**Published:** 2014-01-08

**Authors:** Ruslinda Mustafar, Rozita Mohd, Norazinizah Ahmad Miswan, Rizna Cader, Halim A Gafor, Marlyn Mohamad, Shamsul Azhar Shah, Nor Azmi Kamaruddin, Norella Kong Chiew Tong

**Affiliations:** 1 Department of Medicine, Pusat Perubatan Universiti Kebangsaan Malaysia, Kuala Lumpur, Malaysia; 2 Department of Pathology, Pusat Perubatan Universiti Kebangsaan Malaysia, Kuala Lumpur, Malaysia; 3 Department of Community Health, Pusat Perubatan Universiti Kebangsaan Malaysia, Kuala Lumpur, Malaysia

**Keywords:** Renal Insufficiency, Chronic, Vitamin D, Calcitriol

## Abstract

**Background:**

Hypovitaminosis D (serum 25-OHD < 30 ng/mL) is common in patients with chronic kidney disease (CKD). Vitamin D is believed to involve in the regulation of renin-angiotensin system and may be renoprotective.

**Objectives:**

To compare the effects of calcium with or without calcitriol on renal function in patients with CKD.

**Patients and Methods:**

A prospective randomized trial was performed involving patients with stages 2-4 CKD and hypovitaminosis D. Baseline demographics data were taken at baseline. Patients were randomized equally into oral calcitriol plus calcium carbonate (calcitriol group) or calcium carbonate alone (non-calcitriol group). Serum levels of 25-hydroxyvitamin D (25-OHD), 1,25-dihydroxyvitamin D3 (1,25-(OH)2D), creatinine, calcium and urine protein creatinine index (uPCI) were measured at 6 and 12 weeks.

**Results:**

Fifty (21 Female: 29 Male) patients with CKD with a median age of 53 (22-65) years were recruited. Their median MDRD eGFR (modification of diet in renal disease, estimation of glomerular filtration rate) was 36.0 (15-89) mL/min/1.73 m^2^ with the CKD stage 2 (n = 8, 16%), stage 3 (n = 29, 58%), and stage 4 (n = 13, 26%) respectively. In both study groups serum 25-OHD levels were increased at 12 weeks (P = 0.001), in contrast to serum 1,25-(OH)2D levels which remained unchanged (P > 0.05), serum creatinine and uPCI were also remained unchanged until the end of study (P > 0.05 each). Patients with diabetes had higher serum creatinine (P = 0.01) and lower serum 1,25-(OH)2D (P = 0.02) at baseline. Regardless of the diabetics status, the serum 25-OHD was increased, and 1,25-(OH)2D remained unchanged at 12 weeks in both study groups. At 12 weeks, serum creatinine was decreased in patients with diabetes in the noncalcitriol group (P = 0.03) compared to stabilization of creatinine in the calcitriol group (P > 0.05). Serum calcium was increased, though it was still within the normal range in the calcitriol group (P < 0.001); whereas, in the noncalcitriol group, there was an initial reduction but increased back to baseline (P = 0.007). Urine PCI remained unchanged in both groups.

**Conclusions:**

We have demonstrated that calcitriol supplementation did not offer any additional benefit to reduce 25-OHD and 1,25-(OH)2D levels over calcium carbonate alone in patients with CKD in this short term study. Overall renal function remained unchanged. However, we found that calcitriol at 0.5 mg daily plus calcium carbonate 500 mg daily could be reno-protective in diabetic nephropathy regardless of their serum 25-OHD levels.

## 1. Background

Vitamin D is an important hormone for calcium metabolism and maintenance of bone health, as well as its important role in many chronic diseases including cardiovascular disease (1, 2). Hypovitaminosis D is defined as serum 25-OHD of less than 30 ng/mL which is highly prevalent in the general population and even more common in patients with chronic kidney disease (CKD) (3-5). The major circulating vitamin D metabolite is 25-hydroxyvitamin D [25-OHD], and the 1,25-dihydroxyvitamin D [1,25-(OH)2D] is the active metabolite responsible for the vitamin D actions. The concentration of 1,25(OH)2D or active vitamin D is directly influenced by serum calcium, phosphate and parathyroid hormone (PTH) (6).

Pietro Ravani et al. found that the levels of both 25-OHD and active vitamin D decrease as estimated glomerular filtration rate (eGFR) declines, and 25-OHD is an independent inverse predictor of disease progression and death in patients with CKD stages 2-5 (7). Vitamin D deficiency also predisposes patients with CKD to a high morbidity and mortality due to metabolic bone disease, atherogenesis, insulin resistance and renin-angiotensin-aldosterone system (RAAS) activation leading to chronic renal inflammation and immune dysfunction (8). Chronic inflammation results in scarring of the kidney with persistent proteinuria. Chronic uremia, reduced kidney mass, reduced kidney function or reduced GFR, and protein losses are some of the contributors to hypovitaminosis D in CKD.

Traditionally, therapy with active form vitamin D in patients with CKD is for the prevention and treatment of metabolic bone disease (9, 10). However in the recent years, emerging data suggest that vitamin D plays a role in the regulation of renin-angiotensin system, promotion of vascular endothelial growth factor release, and vascular calcification as well as immune modulation (1, 11). There is a paucity of data regarding the effect of active vitamin D replacement in patients with CKD and hypovitaminosis D. A recent randomized control trial concluded that administration of vitamin D supplements is associated with decrease in overall mortality rate (12, 13). Current practice advocates routine vitamin D prescription only in patients undergoing chronic dialysis for prevention or treatment of secondary hyperparathyroidism (9). Many studies showed that supplementation with ergocalciferol (vitamin D2) or cholecalciferol (vitamin D3) in patients with CKD stages 3-5 is associated with improvement in serum 25-OHD levels (14-16).

## 2. Objectives

We aimed to compare the effect of calcium supplementation with or without calcitriol in patients with CKD stage 2-4 and vitamin D levels (25-OHD) < 30 ng/mL. We hypothesized that calcitriol supplementation in the early stages of CKD may be renoprotective resulting in an increment of serum 25-OHD, stabilization of serum creatinine with no hypercalcemia compared to calcium carbonate alone.

## 3. Patients and Methods

This is a single center open labelled prospective study conducted in the Nephrology Clinic, UKM Medical Centre (UKMMC). The UKM Research Ethics Committee (UKMREC) approved this investigation with the research code number of FF137-2011. It was funded by a research grant of the same center and the Malaysian Society of Nephrology (MSN).

Fifty patients with CKD stages 2 to 4 aged 20 to 65 years with serum 25-hydroxyvitamin D (25-OHD) levels of less than 30 ng/mL were recruited. We excluded patients with acute kidney injury, dialysis dependence, evidence of chronic liver disease and granulomatous diseases such as tuberculosis, sarcoidosis, and malabsorption syndrome. We also excluded patients received any drugs or any health food supplements (either prescribed or over the counter) that could influence vitamin D metabolism. We chose calcitriol 0.5 mcg once a day because it is an active vitamin D and is the lowest minimal dose capable of increasing serum 1, 25-(OH)2D level (17). To ensure adequate absorption of this calcitriol, minimum dose of calcium is needed (17). Therefore, we decided to add 500 mg CaCO3 daily; also, it was given to the other group to minimize confounding factors.

Patients’ demographics data were recorded at baseline prior to equal randomization into either calcitriol group (calcitriol 0.5 mcg + calcium carbonate 500 mg daily) or non-calcitriol group (calcium carbonate 500 mg daily only) and followed for 12 weeks. Patients were instructed not to change their eating habits throughout the study. Angiotensin converting enzyme (ACE) inhibitor and/or angiotensin receptor blocker (ARB) and statin use and dosage were not altered unless medically indicated. The baseline data, serum 25-OHD, 1,25-(OH)2D, calcium, creatinine and urine protein creatinine index (uPCI) were repeated at weeks 6 and 12. Whereas the serum intact PTH was measured at baseline and at the end of study. The dosage and duration of treatment (calcitriol and calcium carbonate) were supplied in exact quantities for the 12-week study period using the usual computerized prescribing system at the UKMMC. Compliance was assessed by asking the patients in the usual way. Blood and urine samples were collected to measure serum calcium, creatinine and uPCI using routine methods available in our center.

### 3.1. Vitamin D Assay

Serum was processed and stored at -20°C to measure 25-OHD and 1,25-(OH)2D. Serum 25-OHD was measured in duplicates using radio immune assay (RIA) method with commercially available kits (DiaSorin, Minnesota, The USA). Serum 1,25-(OH)2D was also measured in duplicates by immunoextraction and quantitation using enzyme immunoassays (EIA) using commercially available kits by diasorin as well.

### 3.2. Statistical Analyses

Statistical analyses were performed using IBM SPSS statistic version 19. All numerical data were subjected to normality testing using Shapiro-Wilk test and expressed as mean ± standard deviation (SD) or median (inter quartile range) for normal and non-normally distributed data. Parametric tests including Student’s t-test was used for normally distributed data; whereas, Mann-Whitney U test was used for non-normally distributed data. The Wilcoxon rank sum test was used within the groups. For qualitative data, Chi-Square test and Fisher’s Exact test were used, and data for each group were compared using ANOVA and Kruskal Wallis test. A P value < 0.05 was considered significant.

## 4. Results

Fifty patients with CKD with a median age of 53 (22-65) years were recruited. Their median MDRD eGFR was 36.0 (15-89) mL/min/1.73 m^2^ with the CKD stage 2 (n = 8, 16%), stage 3 (n = 29, 58%) and stage 4 (n = 13, 26%) respectively. They were randomly assigned into two groups; calcitriol (n = 25) and non-calcitriol (n = 25). Baseline demographic characteristics and laboratory parameters are demonstrated in Table 1. 

**Table 1.** Baseline Demographic and Laboratory Parameters of the Two Study Groups

Characteristic	Non-calcitriol Group (n = 25)	Calcitriol Group (n= 25)	P Value
**Age, mean ± SD, y**	52.0 ± 20.5	55 ± 9.5	0.51
**Gender, No. (%)**			0.39
Male	13 (52)	16 (64)	
Female	12 (48)	9 (36)	
**Race, No. (%)**			0.10
Malay	18 (72)	23 (92)	
Chinese	6 (24)	1 (4)	
Indian	1 (4)	1 (4)	
**BP^a^, mean ± SD, mmHg**			
Systolic	135.0 ± 26.0	127.0 ± 21.5	0.88
Diastolic	75.0 ± 15.0	76.0 ± 13.0	0.63
**Aetiology of CKD^a^, No. (%)**			0.17
Diabetic nephropathy	15 (60)	16 (64)	
Glomerulonephritis	8 (32)	5 (20)	
Hypertension	0 (0)	2 (8)	
Obstructive uropathy	2 (8)	0 (0)	
Others (NSAIDS/ADPKD)	0 (0)	2 (8)	
**Comorbidities, No. (%)**			
Diabetes mellitus	15 (48.4)	16 (51.6)	0.77
Hypertension	21 (45.7)	25 (54.3)	0.11
Dyslipidemia	18 (45)	22 (55)	0.16
Stroke	2 (50)	2 (50)	> 0.99
Ischemic heart disease (IHD)	4 (44.4)	5 (55.6)	> 0.99
**Medications**			
RAAS^a^Blockade (ARB/ACE-I/Spironolactone), No. (%)	15 (44.1)	19 (55.9)	0.23
Calcium channel blocker (CCB), No. (%)	16 (45.7)	19 (54.3)	0.36
β-blocker, No. (%)	11 (42.3)	15 (57.7)	0.26
Statins, No. (%)	18 (48.6)	19 (51.4)	0.75
Serum 25-OHD, median (IQR^a^), ng/mL (NR, > 30)	17.0 (8.05)	15.80 (9.37)	0.44
Serum 1,25-(OH)2D, median (IQR), pg/mL (NR, 39-193)	86.22 (44.91)	91.88 (72.58)	0.96
Serum creatinine, median (IQR), µmol/L (NR, 44-80)	155.0 (87.5)	158.0 (91.0)	0.48
eGFR, median (IQR), mL/min/1.73 m^2^	35.0 (19.0)	36.0 (36.5)	0.29
Urine PCI, median (IQR), g/mmol creatinine (NR, < 0.02)	0.07 (0.12)	0.04 (0.16)	0.32
Serum calcium, mean ± SD, mmol/L (NR, 2.14-2.58)	2.34 ± 0.10	2.30 ± 0.10	0.13

^a^ Abbreviations: ACE-I, angiotensin converting enzyme inhibitor; APKD, adult polycystic kidney disease; ARB, angiotensin receptor blockade; BP, blood pressure; IQR, interquartile range; NSAIDS, non-steroidal anti-inflammatory drugs; RAAS, renin angiotensin aldosterone system.

**Figure 1.**
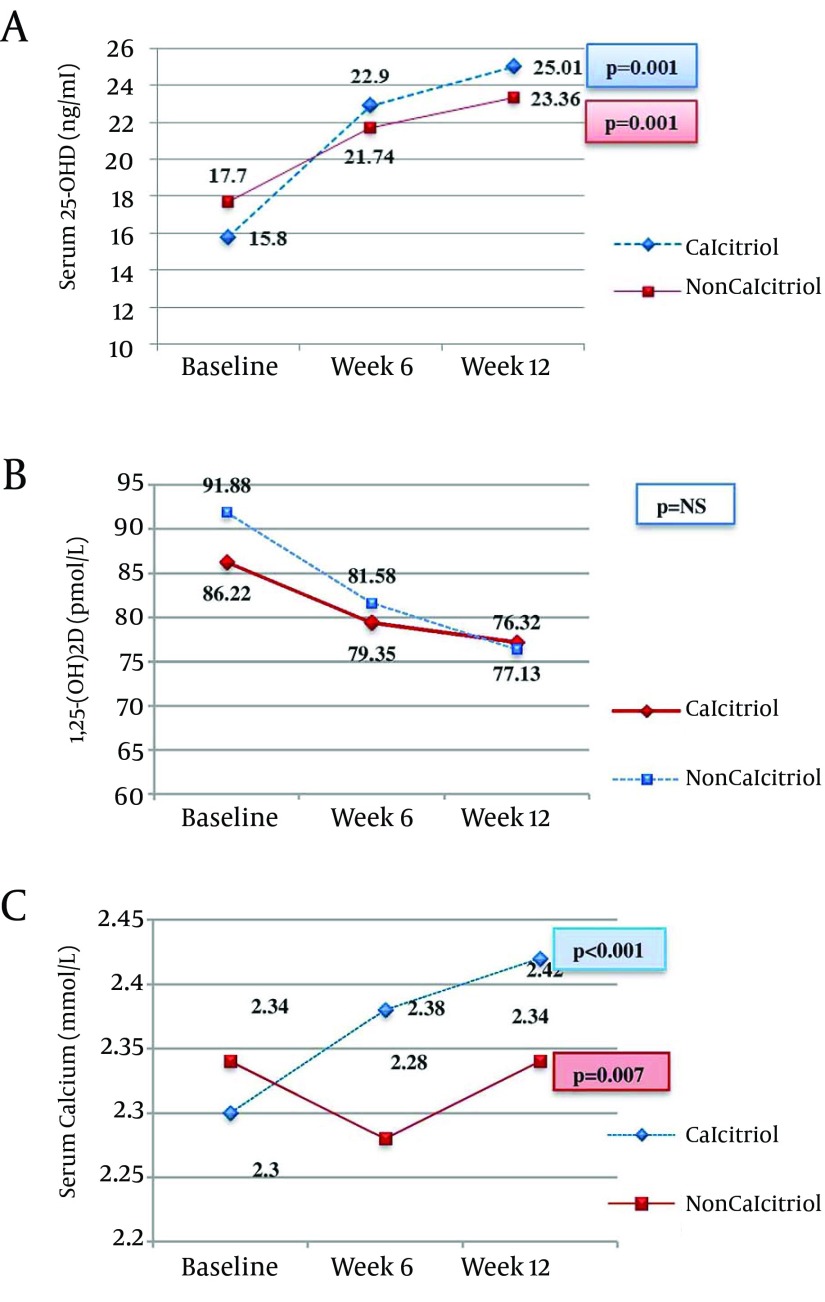
Panel A: Serum 25-OHD, Panel B: Serum 1,25-(OH)2D and Panel C: Serum Calcium in Both Groups Baseline, Week 6 and Week 12.

Figure 1; Panel A showed the serum 25-OHD levels, and indicates that it was significantly increased in both study groups. However when we compare the study groups, all P values were > 0.05 during each visit. Panel B showed serum 1,25-(OH)2D, the P value was not significant for every intragroup and intergroup analysis. Panel C showed the effects on the serum calcium at 6 and 12 weeks in both study groups. The P value between the groups at weeks 6 was 0.003, and at the end of study was 0.052.

The results on serum creatinine, eGFR, urine protein creatinine index (uPCI), iPTH and blood pressure are summarized in Table 2. Further subanalyses among the diabetics and non-diabetics were summarized in Tables 3 and 4.

**Table 2.** Clinical and Laboratory Parameters at Weeks 6 and 12 Post-Supplementation

Parameters	Baseline	Week 6	Week 12	Intra-group P
**Serum creatinine, mean ± SD, µmol/L, (NR, 44-80)**				
Non-calcitriol	155.0 ± 87.5	167.0 ± 103.0	163.0 ± 103.5	0.21
Calcitriol	158.0 ± 91.0	139.0 ± 83.5	165.0 ± 95.0	0.16
Inter group P	0.48	0.37	0.66	
**eGFR^a^, mean ± SD, mL/min/1.73 m^2^**				
Non-calcitriol	35.0 ± 19.0	34.0 ± 21.0	34.0 ± 24.0	0.43
Calcitriol	36.0 ± 36.5	39.0 ± 39.0	32.0 ± 41.5	0.18
Inter group P	0.29	0.17	0.50	
**UrinePCI^a^, mean ± SD, g/mmol creatinine**				
Non-calcitriol	0.07 ± 0.12	0.09 ± 0.15	0.08 ± 0.16	0.54
Calcitriol	0.04 ± 0.16	0.03 ± 0.13	0.05 ± 0.16	0.99
Inter group P	0.32	0.46	0.64	
**iPTH^a^, mean ± SD, pmol/L**				
Non-calcitriol	6.61 ± 6.01	-	5.53 ± 6.53	0.18
Calcitriol	3.57 ± 4.03	-	2.37 ± 3.06	0.25
Inter group P	0.05		0.03	
**Systolic BP^a^, mean ± SD, mmHg**				
Non-calcitriol	135.0 ± 26.0	135.0 ± 20.0	124.0 ± 39.0	0.92
Calcitriol	127 ± 21.5	129.0 ± 26.0	133.0 ± 21.0	0.33
Inter group P	0.88	0.65	0.95	
**Diastolic BP, mean ± SD, mmHg**				
Non-calcitriol	75.0 ± 15.0	74.0 ± 8.0	75.0 ± 14.0	0.98
Calcitriol	76.0 ± 13.0	76.0 ± 11.0	75.0 ± 13.5	0.58
Inter group P	0.63	0.73	0.87	

^a^ Abbreviations: BP, blood pressure; eGFR, estimated glomerular filtration rate; iPTH, intact parathyroid hormone; uPCI, urine protein creatinine index.

**Table 3.** Summarized the Parameters Between Non-diabetics and Diabetics CKD

	Non-diabetics (n = 19)	Diabetics (n = 31)	P Value
**Age, median (IQR^a^), y**	49.0 (20.0)	57.0 (10.0)	0.01
**Gender, No. (%)**			
Male	12 (63.2)	17 (54.8)	0.56
Female	7 (36.8)	14 (45.2)	
**SBP^a^, median (IQR), mmHg**	130.0 (19.0)	132.0 (27.0)	0.29
**DBP^a^, median (IQR), mmHg**	77.0 (15.0)	75.0 (15.0)	0.32
**Medications, No. (%)**			
RAAS^a^ blockade	16 (84.2)	17 (54.8)	0.06
CCB^a^	9 (47.4)	26 (83.9)	0.01
Serum 25-OHD, ng/mL	19.4 (5.9)	14.6 (7.7)	0.05
Serum 1,25(OH)2D	92.5 (55.4)	70.2 (56.5)	0.02
Creatinine, mol/L	120.0 (37.0)	167.0 (63.0)	0.01
eGFR^a^, mL/min/1.73 m^2^	48.0 (37.0)	33.0 (13.0)	0.003
uPCI^a^, g/mmol/creatinine	0.04 (0.07)	0.09 (0.20)	0.10

^a^ Abbreviations: CCB, calcium channel blocker; DBP, diastolic blood pressure; IQR, interquartile range; SBP, systolic blood pressure; RAAS, renin angiotensin aldosterone system; uPCI, urine protein creatinine index.

**Table 4.** Post-supplementation Serum Creatinine, 25-OHD, 1,25-(OH)2D, Urine PCI, Calcium, iPTH and Blood Pressure Among the Non-diabetics and Diabetics Patients With Hypovitaminosis D and CKD^a^

Parameters	Non-diabetic (n = 19, NC = 10, C = 9)			P Value	Diabetic (n = 31, NC = 15, C = 16)			P Value
	Baseline	Week 6	Week 12		Baseline	Week 6	Week 12	
**Serum creatinine, µmol/L, (NR, 44-80)**								
Non calcitriol	122.5 (59.25)	119.5 (64.25)	112.5 (71.75)	0.79	167.0 (65.0)	187.0 (67.0)	194.0 (73.0)	0.03
Calcitriol	107.0 (113.0)	101.0 (122.5)	105.0 (127.5)	0.82	166.0 (67.0)	167.0 (66.75)	184.5 (80.25)	0.14
Inter group P	0.33	0.35	0.39		0.55	0.23	0.62	
**Urine PCI^b^, g/mmol creatinine**								
Non calcitriol	0.06 (0.07)	0.08 (0.14)	0.06 (0.09)	0.46	0.10 (0.14)	0.09 (0.22)	0.11 (0.23)	0.68
Calcitriol	0.03 (0.06)	0.03 (0.08)	0.04 (0.09)	0.53	0.06 (0.34)	0.05 (0.41)	0.06 (0.23)	0.76
Inter group P	0.39	0.43	0.77		0.53	0.74	0.65	
**25-OHD, ng/mL, (NR, > 30)**								
Non calcitriol	19.14 (6.0)	22.8 (8.58)	41.05 (55.33)	0.008	16.19 (8.01)	19.38 (10.61)	21.92 (7.64)	0.01
Calcitriol	19.71 (10.28)	26.34 (10.93)	48.44 (66.16)	0.003	14.43 (13.41)	18.46 (18.0)	19.44 (32.8)	0.001
Inter group P	0.51	0.33	0.87		0.27	0.48	0.58	
**1,25-(OH)** **2D, pmol/L, (NR, 39-193)**								
Non calcitriol	90.42 (30.32)	84.78 (43.99)	90.25 (63.24)	0.50	71.73 (55.28)	75.73 (71.87)	64.87 (37.02)	0.20
Calcitriol	123.64 (70.82)	121.36 (58.97)	171.22 (101.57)	0.61	66.0 (58.55)	71.16 (57.27)	57.43 (57.84)	0.63
Inter group P	0.17	0.18	0.10		0.36	0.58	0.32	
**Serum calcium, mmol/L, (NR, 2.14-2.58)**								
Non calcitriol	2.32 (0.12)	2.27 (0.09)	2.32 (0.10)	0.02	2.39 (0.14)	2.29 (0.22)	2.34 (0.21)	0.28
Calcitriol	2.24 (0.18)	2.28 (0.11)	2.34 (0.22)	0.12	2.33 (0.09)	2.45 (0.14)	2.46 (0.26)	0.19
Inter group P	0.16	0.20	0.84		0.42	0.01	0.03	
**Serum iPTH, pmol/L^b^, pmol/L**								
Non calcitriol	5.84 (4.11)	-	6.34 (8.41)	0.45	8.45 (7.21)	-	3.73 (5.84)	0.02
Calcitriol	3.90 (3.09)	-	2.02 (1.51)	0.01	3.29 (4.85)	-	2.72 (9.44)	0.83
Inter group P	0.51		0.003		0.05		0.58	
**SBP^b^, mmHg**								
Non calcitriol	129.5 (27.6)	124.0 (12.0)	121.0 (19.6)	0.29	139.0 (24.0)	142.0 (23.0)	151.0 (30.0)	0.42
Calcitriol	130.0 (20.0)	139.0 (20.5)	133.0 (18.0)	0.58	125.5 (27.3)	127.5 (26.0)	134.0 (24.3)	0.37
Inter group P	0.68	0.01	0.05		0.07	0.06	0.06	
**DBP^b^, mmHg**								
Non calcitriol	78.0 (15.5)	76.0 (7.0)	75.0 (13.3)	0.41	74.0 (21.0)	74.0 (12.5)	76.0 (15.0)	0.42
Calcitriol	77.0 (16.0)	80.0 (14.5)	79.0 (14.0)	0.46	75.5 (14.0)	72.5 (12.5)	73.5 (14.6)	0.81
Inter group P	0.44	0.14	0.14		0.95	0.61	0.35	

^a^ Data are expressed as median (IQR).

^b^ Abbreviations: DBP, diastolic blood pressure; iPTH, intact parathyroid hormone; PCI, protein creatinine index; SBP, systolic blood pressure.

## 5. Discussion

It is now proven widely that vitamin D deficiency predisposes patients with CKD to a high morbidity and mortality rate due to metabolic bone disease, atherogenesis, insulin resistance, and renin-angiotensin-aldosterone system (RAAS) activation which leads to chronic renal inflammation and immune dysfunction (8). A previous study in nephrectomized rats showed that vitamin D analog and ACEI therapy resulted in an additional reno-protective effect presumably by regulating the RAAS system (18). Recent data showed that vitamin D is involved in the regulation of renin-angiotensin system (8, 19). Therefore, it seems that vitamin D supplementation possibly improves the kidney function.

Various data have demonstrated that vitamin D and its analogues were able to increase serum 25-OHD even though levels may not be normalized (20, 21). In our study, supplementation with calcitriol + CaCO3 demonstrated an increment in the serum 25-OHD levels at 12 weeks which is in agreement with other studies (20). Pesenson et al. found only slight increment in vitamin D levels in 45 patients with CKD treated with ergocalciferol (vitamin D3) for 6 months (22).

The increment in the serum 25-OHD levels in the calcitriol group can be explained due to the rise in serum calcium which in turn leads to a reduction in iPTH levels; thus, less conversion of serum 25-OHD to the active form 1,25-(OH)2D. Hence, elevation of serum 25-OHD and reduction in serum 1,25-(OH)2D were detected; although, the latter is not statistically significant in this study. Furthermore conversion of 25-OHD to this active form 1,25-(OH)2D lies on the presence of 1-αhydroxylase enzyme from the kidney, therefore no significant change of 1,25-(OH)2D levels was seen in this study regarding the study population are mainly patients with CKD. Interestingly, serum 25-OHD levels were increased in patients of the Non-calcitriol group who were given CaCO3 only. Thus we postulate using the similar concept as mentioned earlier, a rise in serum calcium at the end of study period could contribute to the rise in the serum 25-OHD via its inhibition in iPTH levels, thus less conversion of the 25-OHD to 1,25-(OH)2D. Therefore, supplementation with calcitriol plus CaCO3 caused a rise in serum 25-OHD at a higher magnitude compared to CaCO3 alone with no significant change seen in the serum 1,25-(OH)2D. 

For the direct effects of calcitriol as active vitamin D, no significant change was seen in the serum 1,25-(OH)2 D levels due to the given dose which is still not high enough to have such effects. Wissing et al. showed that patients treated with calcium plus vitamin D3 (cholecalciferol) had significantly higher 25-OHD but not 1,25(OH)2D levels (23). It would require higher pharmacological doses of Vitamin D3 to achieve normalization and maintained serum 25-OHD above 30 ng/mL as demonstrated by Halloran et al. (21).

Previous studies on the effects of supplementation of vitamin D in non-dialyzed CKD patients showed a conflicting results with some studies demonstrating a rise in serum creatinine (24, 25). We found no significant differences in serum creatinine and eGFR at 12 weeks supplementation in both groups which concurs with others (26-28). The stabilization of serum creatinine in both groups could also be contributed by optimal BP control throughout the study period. Whereas their urine PCI in both groups did not change after 12 weeks of supplementation as most of the patients were already on RAAS blockade and their baseline urine PCI was already low.

Diabetic kidney disease (62%) is the leading cause of CKD in our study cohort and is in keeping with local data whereby diabetic kidney disease accounts for 55% of new dialysis patients (29). We found that diabetics subgroup (DM) were older with a lower eGFR and higher serum calcium but no difference in serum iPTH and urine PCI levels. They had a trend towards lower levels of serum 25-OHD and their serum 1,25-(OH)2D levels were also significantly lower as compared to non-diabetics counterpart. These results were consistent with other studies (4, 30-32). Only 54.8% of our patients with diabetes and CKD were on RAAS blockade. The degree of hypovitaminosis D is greater in the diabetic subgroup compared to the non DM and is in keeping with other studies (31, 32). Mehrotra et al. in a post hoc analysis of 146 non-dialyzed patients with diabetes and CKD, found that more than 80% of these patients had suboptimal 25-OHD levels with 38% deficient (< 15 ng/mL) and 45% insufficient (15-30 ng/mL) (4).

In our study, both calcitriol and the non-calcitriol groups had a rise in serum 25-OHD levels regardless of their diabetic status. However, the diabetics had a lower serum 25-OHD levels compared to the non-diabetics, and the increment post supplementation was lower than the non-diabetics. Alshayeb et al. demonstrated that the presence of diabetes and CKD were associated with resistance to a correction of 25-OHD deficiency and they required higher doses of vitamin D to achieve similar increases in 25-OHD than the non-diabetic counterpart (33).

Despite remarkable increment of serum 25-OHD levels post supplementation in this study, it does not influence the serum 1,25-(OH)2D levels. Throughout the 12 week study period, there were no significant changes in the serum 1,25-(OH)2D levels even though with the added calcitriol supplementation.

In the non-calcitriol group of the DM subgroup a rise in the serum creatinine was seen whereas in the calcitriol group, we noted stabilization of serum creatinine. This may be attributed due to the renoprotective property of the vitamin D through its regulation of the RAAS, particularly renin (34, 35). The stabilization of serum creatinine in the DM group was independent of the blood pressure levels. We found no difference in the blood pressure that could be the cause of decrease in the serum creatinine in the non-calcitriol DM group. Their serum calcium levels were within the normal range as well.

Despite emerging data suggesting reno-protective effects of vitamin D in reducing proteinuria (36), in our study no significant change was seen in the proteinuria as determined by urine protein creatinine index methods regardless of their diabetes status as well. Furthermore, our study patients had lower urine PCI levels at their baseline.

The strength of our study is its randomized controlled design with a similar proportion of diabetic patients. Although 3 months follow up may not be sufficient to show the effects on the renal function, we found no deleterious effects especially hypercalcemia at the prescribed dose. However further studies are recommended involving larger population and longer duration.

We demonstrated that the given dose of calcitriol supplementation, did not offer any additional benefit in increasing 25-OHD and 1,25-(OH)2D levels over calcium carbonate alone in patients with CKD within the short term 12 week period. Overall renal function remained unchanged. However, we found that calcitriol at 0.5 mg daily plus calcium carbonate 500 mg daily could be reno-protective in diabetic nephropathy regardless of their serum 25-OHD.
